# Prediction of recurrent gestational diabetes mellitus: a retrospective cohort study

**DOI:** 10.1007/s00404-022-06855-z

**Published:** 2023-01-03

**Authors:** Stephan Hahn, Sabine Körber, Bernd Gerber, Johannes Stubert

**Affiliations:** Department of Obstetrics and Gynecology, Rostock University Medical Center, Suedring 81, 18059 Rostock, Germany

**Keywords:** Gestational diabetes mellitus, Predictive model, Risk of recurrence, Family history, BMI, Obesity, Overweight, Insulin treatment

## Abstract

**Background:**

Women after gestational diabetes mellitus (GDM) are at increased risk for development of GDM recurrence. It was the aim of our study to evaluate factors for prediction of risk of recurrence.

**Methods:**

In this retrospective cohort study we included 159 women with GDM and a subsequent pregnancy. Putative risk factors for GDM recurrence were analyzed by logistic regression models. Results were compared to a cohort of age-matched women without GDM as controls (*n* = 318).

**Results:**

The overall risk of GDM recurrence was 72.3% (115/159). Risk factors of recurrence were a body mass index (BMI) ≥ 30 kg/m^2^ before the index pregnancy (odds ratio (OR) 2.8 [95% CI 1.3–6.2], *p* = 0,008), a BMI ≥ 25 kg/m^2^ before the subsequent pregnancy (OR 2.7 [95% CI 1.3–5.8]. *p* = 0.008), a positive family history (OR 4.3 [95% CI 1.2–15.4], *p* = 0.016) and insulin treatment during the index pregnancy (OR 2.3 [95% CI 1.1–4.6], *p* = 0.023). Delivery by caesarean section (index pregnancy) was of borderline significance (OR 2.2 [95% CI 0.9–5.2], *p* = 0.069). Interpregnancy weight gain, excessive weight gain during the index pregnancy and fetal outcome where not predictive for GDM recurrence. Neonates after GDM revealed a higher frequency of transfer to intensive care unit compared to healthy controls (OR 2.3 [95% CI 1.1–4.6], *p* = 0.0225). The best combined risk model for prediction of GDM recurrence including positive family history and a BMI ≥ 25 kg/m^2^ before the subsequent pregnancy revealed moderate test characteristics (positive likelihood ratio 7.8 [95% CI 1.1–54.7] and negative likelihood ratio 0.7 [95% CI 0.6–0.9]) with a positive predictive value of 96.6% in our cohort.

**Conclusions:**

A positive family history of diabetes mellitus in combination with overweight or obesity were strongly associated with recurrence of a GDM in the subsequent pregnancy. Normalization of the pregravid BMI should be an effective approach for reducing the risk of GDM recurrence.

## What does this study add to the clinical work

**Table:** 

A positive family history of diabetes mellitus and a high pregravid body mass index were independent risk factors of recurrent gestational diabetes mellitus. In contrast, interpregnancy weight gain and excessive weight gain during pregnancy were not related to the risk of recurrence. Normalization of pregravid body mass index before the next pregnancy can reduce the risk of recurrence.	

## Introduction

Gestational diabetes mellitus (GDM) is defined as any kind of glucose intolerance with onset or first recognition during pregnancy [1]. GDM increases the risk of an adverse pregnancy outcome including gestational hypertension, neonatal macrosomia or shoulder dystocia, but is also associated with development of a metabolic syndrome and a type 2 diabetes mellitus later in life [2–5]. The German guideline recommends a screening via 75 g oral glucose tolerance test between 24 + 0/7 and 27 + 6/7 weeks of gestation [6]. But it remains a great challenge to identify women with a high risk for GDM as early as possible, because an early start of lifestyle interventions, periconceptional or during the first trimester, seemed to be the most effective intervention for an avoidance of pregnancy complications [7, 8].

Women with a history of a GDM in a previous pregnancy are at high risk for developing a GDM recurrence in a subsequent pregnancy [8] and would be ideal candidates for early lifestyle intervention or treatment initiation for improvement of outcome [3, 9].

It was the aim of this retrospective analysis to obtain data on prevalence of GDM recurrence and to identify predictive risk factors.

## Methods

### Recruitment of patients and definitions

**Fig. 1:**
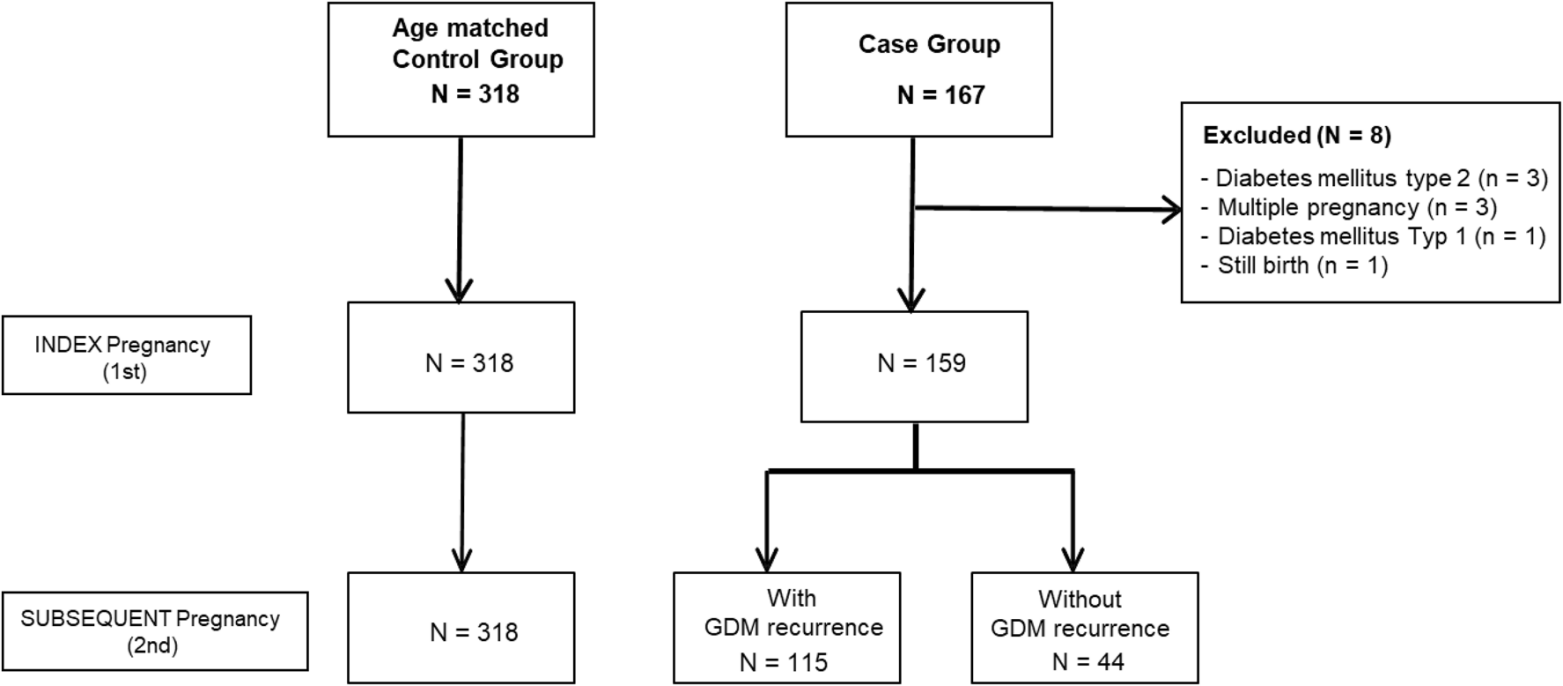
Flowchart of patient’s selection with criterions of exclusion

This is a single center retrospective cohort analysis on a German tertiary care center. We searched for all women with a GDM (index pregnancy), who had a subsequent pregnancy with a viable newborn. The record screening was performed digitally by searching for the International Classification Disease (ICD-10) code O24.4 (Fig. 1). Criterions of exclusion were multiple pregnancies, diagnosis of a diabetes mellitus type 1 or 2, still birth or an abortion. Between January 2014 and September 2020, a total of 159 women were included. A group of 318 women with two pregnancies during the study period but without GDM served as healthy control group. The women of the control group were matched by the maternal age at the first and the subsequent pregnancy.

A positive family history was defined as any diabetes mellitus diagnosis of parents or siblings. Women’s weight before the first pregnancy was subtracted from that before the second pregnancy to compute the interpregnancy weight gain (IWG). Interpregnancy interval means the time between the delivery of the index pregnancy and the beginning of the subsequent pregnancy. Neonatal birth weight centiles were calculated in accordance to the German growth charts and depended on neonatal sex, birth weight and age at birth [10]. Neonatal macrosomia was considered a birth weight ≥ 90th centile. Preterm birth was defined as delivery before 37 weeks of gestational age. Depending on pregravid body mass index (BMI) the women were classified to underweight (BMI ≤ 18.5 kg/m^2^), normal weight (BMI 18.5–24.9 kg/m^2^), overweight (BMI 25.0–29.0 kg/m^2^) and obese (BMI ≥ 30.0 kg/m^2^). For these groups we identified excessive weight gain during pregnancy based on the IOM definition [11].

### Statistical analysis

Statistical analysis of data was made using IBM SPSS statistics package 27.0 (SPSS Inc. Chicago, IL, USA) and Excel 2016 (Microsoft Corporation, Redmond, WA, USA). The normality of data was assessed by Shapiro–Wilk test and Q-Q-plot analysis. For between group comparisons we used the Student’s *t* test, the Mann–Whitney *U* test or, for comparison of more than two groups, the Kruskal–Wallis test, as appropriate. Categorical data were evaluated by Chi-square test or Fisher's exact test and diagnostic odd ratios (OR) with 95% confidence interval (CI) were presented if available. All *p*-values were obtained using two-sided statistical tests, and values < 0.05 were considered statistically significant.

A logistic regression model was used to assess the independence of specific risk parameters and to compute a combined predictive risk model for GDM recurrence. The following risk factors were included: positive family history, pregravid BMI ≥ 30 kg/m^2^ or ≥ 25 kg/m^2^ for the index and subsequent pregnancy, insulin treatment during index pregnancy, and delivery by caesarean section (index pregnancy). Receiver operating characteristics (ROC) curves and the area under the curves (AUC) were computed using the combined risk models. Based on the results of the logistic regression models we verified the test characteristics for three predictive models of combined risk factors: positive family history and obesity before index pregnancy (model 1), positive family history and BMI ≥ 25 kg/m^2^ before subsequent pregnancy (model 2). For model 3 we added the risk factor caesarean section at index pregnancy to the risk factors of model 2.

The local ethics committee does not request formal approval for anonymized retrospective analysis of clinical data.

## Results

### Patients’ characteristics

**Fig. 2:**
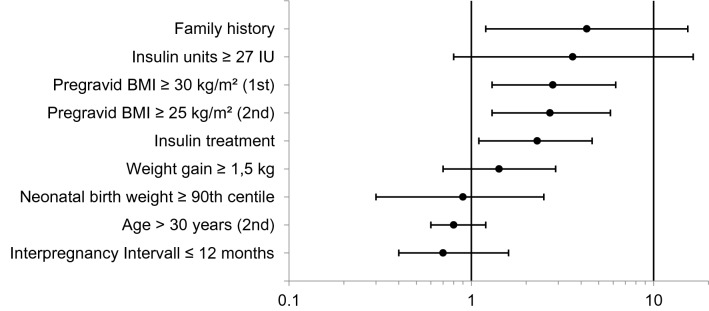
Risk factors of recurrence of gestational diabetes mellitus. Forest Plot shows unadjusted odds ratios (black dot) with 95% confidence interval (whisker)

After GDM during first pregnancy, 115 of 159 women (72.3%) developed a GDM recurrence in the subsequent pregnancy. Basal patient’s characteristics did not differ between groups in respect to maternal age, previous births and the time interval between the pregnancies (Table 1). Patients with GDM recurrence showed a higher pregravid BMI during the index pregnancy as well as the subsequent pregnancy. Obesity before the index pregnancy (OR 2.8 [95% CI 1.3–6.2], *p* = 0,008) was a risk factor for GDM recurrence (Table 1, Fig. 2). However, the difference was not significant in case of obesity before the subsequent pregnancy (OR 1.9 [95% CI 0.9–3.8], *p* = 0.087). Nevertheless, the risk of GDM recurrence also increased with increasing BMI before the subsequent pregnancy with a lower cut-off of BMI ≥ 25 kg/m^2^ (OR 2.7 [95% CI 1.3–5.8]. *p* = 0.008). IWG did not differ between groups. Women with recurrent GDM revealed a higher prevalence of a positive family history of diabetes (OR 4.3 [95% CI 1.2–15.4], *p* = 0.016) and needed more frequently an insulin treatment (OR 2.3 [95% CI 1.1–4.6], *p* = 0.023). In contrast, the required insulin dosage and the weight gain during the index pregnancy did not differ between patients with and without GDM recurrence. Delivery by caesarean section was in trend more common in women with GDM recurrence (OR 2.2 [95% CI 0.9–5.2], *p* = 0.069).

**Table 1:** Characteristics of patients with gestational diabetes mellitus (GDM)

	Gestational diabetes mellitus		*p*-value
	With recurrence *n* = 115	w/o recurrence *n* = 44	
**Index pregnancy**			
Maternal age, y	29.5 ± 4.7	30.3 ± 4.5	0.352
Family history of diabetes, *n* (%)	34 (29.6)	3 (6.8)	**0.017**
Pregravid BMI, kg/m^2^	29.5 (24.5–34.8)	25.3 (22.4–31.4)	**0.017**
Pregravid BMI ≥ 25 kg/m^2^, *n* (%)	83 (72.2)	25 (56.8)	0.063
Pregravid BMI ≥ 30 kg/m^2^, *n* (%)	56 (48.7)	11 (25.0)	**0.007**
Gravidity, *n*	2 (1–3)	2 (1–2.8)	0.615
Parity, *n*	1 (1–2)	1 (1–2)	0.813
Gestational age at delivery, weeks	39 (38–40)	39 (38.3–40)	0.488
Caesarean section, *n* (%)	38 (33.0)	8 (18.2%)	0.079
Insulin treatment, *n* (%)	75 (65.2)	20 (45.5)	**0.030**
Relative max. Insulin dose (IE/kg)	0.23 (0.15–0.31)	0.23 (0.18–0.30)	0.892
Max. insulin dose per day, IE	20 (14–34)	20 (18–26)	0.906
Umbilical artery pH	7.26 ± 0.06	7.28 ± 0.09	0.182
APGAR 5 min	10 (9–10)	10 (9–10)	0.470
Birth weight, *g*	3540 ± 471	3470 ± 467	0.401
Birth weight centile	59 (36–79)	43 (27–77.8)	0.204
Min. neonatal blood glucose, mmol/l	2.9 ± 0.6	3.0 ± 0.7	0.849
NICU, *n* (%)	10 (8.7)	7 (15.9)	0.250
Weight gain during pregnancy, kg	12 (7–17)	13.5 (8.3–18.8)	0.162
Excessive weight gain, *n* (%)	54 (47.0)	22 (50.0)	0.803
**Subsequent pregnancy**			
Maternal age, y	32.1 ± 4.7	32.9 ± 4.9	0.338
Pregravid BMI (kg/m^2^)	31.1 (25.6–36.6)	26.6 (22.0–34.4)	**0.009**
Pregravid BMI ≥ 25 kg/m^2^, *n* (%)	90 (78.3)	25 (56.8)	**0.007**
Pregravid BMI ≥ 30 kg/m^2^, *n* (%)	62 (53.9)	17 (38.6)	0.085
Interpregnancy weight gain, kg	3 (− 1 to 8)	1 (− 3 to 7)	0.300
Time between pregnancies, months	22 (11–29)	21.5 (11.3–27.8)	0.810
Gestational age at delivery, weeks	39 (38–39)	39 (38–40)	**0.012**
Caesarean section, *n* (%)	45 (39.1)	10 (22.7)	0.063
Umbilical artery (pH)	7.28 ± 0.08	7.28 ± 0.08	0.722
APGAR 5 min	10 (9–10)	10 (9–10)	0.603
Birth weight, *g*	3673 ± 525	3543 ± 680	0.201
Birth weight centile	72 (50–91)	67.5 (35–83.3)	0.103
NICU, *n*	14 (12.2)	2 (4.5)	0.238
Weight gain during pregnancy, kg	10 (6–14)	12.5 (8–16.8)	**0.041**
Excessive weight gain, *n* (%)	40 (34.8)	18 (40.9)	0.581

Patients with and without GDM recurrence were compared

Data are presented as mean ± standard deviation, median with interquartile range or with absolute and relative frequencies

Neonatal outcome was similar between groups in the index as well as the subsequent pregnancy even if gestational age at delivery was marginal earlier in the subsequent pregnancy in patients with GDM recurrence, but without impact on the rate of preterm birth. Women with GDM recurrence showed a trend to higher birth weight centiles. A comparison of median birth weight centiles between women with GDM recurrence, women without GDM recurrence and controls revealed a continuous decrease during the index pregnancy (59; 43 and 49; *p* = 0.012) as well as the subsequent pregnancy (72; 67.5 and 58, *p* < 0.001).

Compared to the age-matched controls, women with GDM in the index pregnancy showed a higher pregravid BMI (Table 2). Prevalence of obesity increased from control cohort (10.7%) to GDM patients without recurrence (25.0%) and was highest in the group of patients with GDM recurrence (48.7%, *p *< 0.001).

**Table 2:** Comparison of characteristics of patients with gestational diabetes mellitus (GDM) and unaffected, age-matched women as controls (CTRL)

	GDM	CTRL	*p*-value
	*n* = 159	*n* = 318	
**Index pregnancy**			
Maternal age, y	29.8 ± 4.6	29.8 ± 4.6	0.944
Pregravid BMI, kg/m^2^	28.3 (23.9–34.7)	23.2 (20.8–25.9)	** < 0.001**
Gravidity, *n*	2 (1–3)	2 (1–3)	0.536
Parity, *n*	1 (1–2)	1 (1–2)	0.508
Gestational age at delivery, weeks	39 (38–40)	39 (38–40)	0.183
Umbilical artery pH	7.27 ± 0.073	7.28 ± 0.074	**0.040**
APGAR 5 min	10 (9–10)	10 (9–10)	0.085
Birth weight, *g*	3521 ± 479	3399 ± 525	**0.014**
Birth weight centile	56 (32–79)	48 (25–74)	**0.007**
NICU, *n* (%)	17 (10.7%)	16 (5.0)	**0.034**
Weight gain during pregnancy, kg	12 (8–17)	14 (11–19)	** < 0.001**
Excessive weight gain, *n* (%)	76 (48.4)	144 (45.3)	0.625
Caesarean section, *n* (%)	46 (28.9)	66 (20.8)	0.052

	GDM w/o recurrence	CTRL	*p*-value
	*n* = 44	*n* = 318	
**Subsequent pregnancy**			
Maternal age, y	32.9 ± 4.9	32.4 ± 4.6	0.499
Pregravid BMI (kg/m^2^)	26.6 (22.0–34.4)	23.7 (21.3–27.6)	**0.006**
Weight gain during pregnancy, kg	12.5 (8–16.8)	13.5 (10–18)	0.127
Interpregnancy weight gain, kg	1 (− 3 to 7)	2 (− 1 to 6)	0.555
Time between pregnancies, months	21.5 (11.3–27.8)	21 (12–30)	0.605
Gestational age at delivery, weeks	39 (38–40)	39 (38–40)	0.967
Umbilical artery (pH)	7.28 ± 0.078	7.29 ± 0.076	0.996
APGAR 5 min	10 (9–10)	10 (9–10)	0.299
Birth weight, *g*	3543 ± 680	3499 ± 608	0.659
Birth weight centile	67.5 (35–83.3)	58 (33.8–79.3)	0.550
NICU, *n*	2 (4.5)	19 (6.0)	0.761
Excessive weight gain, *n* (%)	18 (40.9)	141 (44.3)	0.747
Caesarean section, *n* (%)	10 (22.7%)	62 (19.5%)	0.687

Data are presented as mean ± standard deviation, median with interquartile range or with absolute and relative frequencies

Even the maternal weight gain of women with GDM was lower during pregnancy, the median birth weight and the median birth weight centiles of the newborns were higher compared to controls without GDM (Table 2). Admission to neonatal intensive care unit (NICU) was more frequently required after a pregnancy with GDM (OR 2.3 [95% CI 1.1–4.6], *p* = 0.0225).

### Predictive models for GDM recurrence

The risk factors for GDM recurrence were further analyzed by logistic regression creating three predictive models (Table 3). In all models, a positive family history and a higher BMI where independent risk factors of GDM recurrence, whereas the need of an insulin treatment during the index pregnancy depends on family history and the addition did not further improved the models. Delivery by caesarean section was of borderline significance and was therefore included in the regression analysis, but did also not improve the model. The predictive models resulted in ROC-AUC values of maximum 0.72 (Table 4). The test characteristics for the combination of a positive family history with a BMI ≥ 25 kg/m^2^ before the subsequent pregnancy (OR 10.6 [95% CI 1.4–81.5], *p* = 0.024) were superior to the combination of the family history with a BMI ≥ 30 kg/m^2^ before the index pregnancy (OR 6.3 [95% CI 0.8–49.7], *p* = 0.079) (Table 5).

**Table 3:** Risk factors for recurrence of gestational diabetes mellitus (GDM)

Risk factor (predictor)	% GDM recurrence with risk factor	% GDM recurrence without risk factor	Unadjusted OR (95% CI)	*p* value	Model 1		Model 2		Model 3	
					Adjusted OR (95% CI)	*p* value	Adjusted OR (95% CI)	*p* value	Adjusted OR (95% CI)	*p* value
Family history of diabetes	91.9	72.4	4.3 (1.2–15.4)	**0.024**	3.8 (1.0–13.7)	**0.044**	4.3 (1.2–15.8)	**0.027**	4.4 (1.2–16.4)	**0.025**
Pregravid BMI ≥ 25 kg/m^2^ (index pregnancy)	76.9	62.7	2.0 (1.0–4.1)	0.066						
Pregravid BMI ≥ 30 kg/m^2^ (index pregnancy)	83.6	64.1	2.8 (1.3–6.2)	**0.008**	3.0 (1.1–8.1)	**0.035**				
Pregravid BMI ≥ 25 kg/m^2^ (subsequent pregnancy)	78.3	56.8	2.7 (1.3–5.8)	**0.008**			3.3 (1.3–8.5)	**0.016**	3.1 (1.2–7.9)	**0.021**
Pregravid BMI ≥ 30 kg/m^2^ (subsequent pregnancy)	78.5	66.3	1.9 (0.9–3.8)	0.087						
Insulin treatment (index pregnancy)	78.9	62.5	2.3 (1.1–4.6)	**0.025**	1.3 (0.5–3.2)	0.586	1.0 (0.4–2.6)	0.958		
Caesarean section (index pregnancy)	82.6	68.1	2.2 (0.9–5.2)	0.069					2.7 (0.8–8.9)	0.098

Model 1 including family history, BMI ≥ 30 kg/m^2^ in index pregnancy and insulin treatment

Model 2 including family history, BMI ≥ 25 kg/m^2^ in subsequent pregnancy and insulin treatment

Model 3 including family history, BMI ≥ 25 kg/m^2^ in subsequent pregnancy and caesarean section in index pregnancy

Unadjusted and adjusted Odds ratios (OR) with 95% confidence interval (CI) are presented

Adjustment was performed by logistic regression analysis

**Table 4:** Prediction of recurrence of gestational diabetes mellitus by combined risk models

Predictive model	ROC-AUC value	95% CI	*p*-value
Family history + obesity (1st) + insulin treatment	0.700	0.599–0.802	0.000
Family history + overweight (2nd) + insulin treatment	0.665	0.555–0.774	0.003
Overweight (2nd) + insulin treatment	0.631	0.527–0.734	0.013
Obesity (1st) + insulin treatment	0.670	0.574–0.767	0.001
Family history + obesity (1st)	0.692	0.587–0.798	0.000
Family history + overweight (2nd)	0.690	0.583–0.797	0.001
Family history + overweight (2nd) + caesarean section (1st)	0.722	0.621–0.823	0.000

Receiver operating characteristics (ROC) analysis with area under the curve (AUC) for various combined predictive models

Predictive probabilities of the different models where computed by a logistic regression model

1st = index pregnancy, 2nd = subsequent pregnancy

**Table 5:** Test characteristics of predictive models

Predictive model	Sensitivity	Specificity	PPV	NPV	Accuracy	LR +	LR−
Family history + obesity (1st)	19.6	96.3	95.0	25.0	36.3	5.3 (0.7–37.7)	0.8 (0.7–0.9)
Family history + overweight (2nd)	28.9	96.3	96.6	27.4	43.5	7.8 (1.1–54.7)	0.7 (0.6–0.9)
Family history + overweight (2nd) + caesarean section (1st)	11.3	100	100	23.9	30.7	n.a	0.9 (0.8–1.0)

*LR* likelihood ratio

## Discussion

This study examined retrospectively influencing factors on GDM recurrence in a subsequent pregnancy. We demonstrated that a positive family history of diabetes, overweight or obesity and the need for insulin treatment are associated with GDM recurrence. The multiple logistic regression analysis revealed a positive family history of diabetes and a pregravid BMI ≥ 25 kg/m^2^ before the subsequent pregnancy as the strongest independent predictors of GDM recurrence.

In our cohort, 72.3% of women with GDM had a recurrence in the subsequent pregnancy, what is at the upper range value. In a systematic review the recurrence rates varied between 30 and 84% [12]. Reasons for a high recurrence rate are assumed to be the ethnical origin including differences in lifestyle factors like nutrition practice and maternal age [9, 13]. In our study cohort a western nutrition predominates with a tendency to overweight and obesity. For example, the mean pregravid BMI of women with GDM recurrence in a Chinese study was 22.8 kg/m^2^ (vs. 30.4 kg/m^2^ in our cohort) with a recurrence rate of 55% [14]. A Scandinavian population-based cohort study including 4078 women with GDM in their first pregnancy over a period of 22 years (1992–2014) showed an overall recurrence risk of 39% [15]. Even if the recurrence rate increased to 43.6% for women with a BMI ≥ 25 kg/m^2^, the risk of recurrence remained low, which may be affected by differences in the GDM definition and screening strategies [16]. It is well known, that the detection rate of GDM essentially depends on the used glucose tolerance test [17]. In Germany, the GDM diagnosis is based on a 75 g oral glucose tolerance test in accordance to the IADPSG guideline [1]. The test had the highest sensitivity compared to other test strategies [17]. These differences impede a direct comparison of recurrence rates.

The pregravid BMI was a risk factor during the index as well as the subsequent pregnancy, which is in accordance with others [14, 15, 18]. Most studies also observed a positive correlation between IWG and risk of GDM recurrence [2, 15, 18, 19], but in our study existed only a trend to an increased risk and was therefore of less impact compared to the pregravid BMI. Compared to others the IWG was lower in our study cohort and did not differ between women with GDM and women from the unaffected control group [15, 20].

Weight gain during pregnancy as well as an excessive weight gain in accordance to the IOM definition was not associated with an increased risk of GDM recurrence, confirming the results of a recent retrospective study [21].

A positive family history of diabetes mellitus is a well-known risk factor for development of GDM [23–25] and was also associated with an increased risk of GDM recurrence [2, 26]. This association may not only based on genetic but also on non-genetic environmental components [23, 24]. In our study the positive family history was the strongest predictor of GDM recurrence and thereby was independent from BMI. Insulin treatment was associated with increased risk for GDM recurrence in our as well other studies [16, 18, 27]. However, in the logistic regression analysis this risk factor failed to be independent after adjusting for family history pointing out the probability of a genetic association between family history and insulin resistance.

Although an increase in maternal age was regarded as a risk factor for GDM recurrence in several studies, we were not able to confirm this association in our study [2, 18, 20, 22].

Neonatal factors like birth weight, levels of blood glucose or stay at neonatal intensive care unit (NICU) where not predictive for GDM recurrence. However, newborns of women with GDM revealed higher birth weight, higher birth weight centiles and were more frequent on NICU compared to unaffected women.

Donovan et al. [28] and Zheng et al. [29] developed predictive models for development of GDM in nulliparous women, which included, among others, family history and pre-pregnancy BMI as strong risk factors and thereby are consistent with our predictive parameters for GDM recurrence. In cases of a positive family history and a BMI ≥ 25 kg/m^2^ before the subsequent pregnancy more than 95% of women developed a recurrent GDM. However the other test characteristics were only moderate which is in accordance to the ROC-AUC of nearly 0.7.

Generally, our study is limited by the small number of cases, discovering only risk factors of high impact, but therefore of clinical relevance. Additionally, due to the retrospective design, we were not able to receive complete data of some putative predictive parameters of GDM recurrence like the values of the 75-g oral glucose tolerance test in the index pregnancy. After a pregnancy with GDM it is recommend to exclude a diabetes mellitus type 2 by a 75-g oral glucose tolerance test six to twelve weeks after delivery [6, 26], because there is a relative risk between 7.4 and 8.9 to develop a diabetes mellitus type 2 later in life [30, 31]. In our study it was not reproducible, if this test took place. In a German prospective cohort study, 4% of women revealed a diabetes mellitus type 2 in the postpartum screening with a 75-g oral glucose tolerance test, 48% showed an impaired glucose tolerance and/or impaired fasting glucose level [32]. However, the postpartum screening rates are internationally low [33, 34].

## Conclusion

In conclusion, a positive family history of diabetes as well as overweight and obesity are strongly associated with recurrence of GDM. Whereas the family history is a non-modifiable risk factor, the normalization of the BMI is in principle feasible and may modulate the risk of GDM recurrence. Effective health care programs for weight reduction in women with overweight and obesity after GDM, especially if associated with a positive family history, should be therefore recommend, but may be difficult to implement.

## Data Availability

The data that support the findings of this study are available from the corresponding author upon reasonable request.

## Abbreviations

AUC: Area under curve
BMI: Body mass index
CI: Confidence interval
GDM: Gestational diabetes mellitus
IWG: Interpregnancy weight gain
LR: Likelihood ratio
NICU: Neonatal intensive care unit
OR: Odds ratio
ROC: Receiver operating characteristic
